# Effect of Transglutaminase-Mediated Cross-Linking on Physicochemical Properties and Structural Modifications of Rice Dreg Protein

**DOI:** 10.3390/foods14213719

**Published:** 2025-10-30

**Authors:** Xianxin Chen, Xiaoyan Zhu, Fangjian Ning, Songyu Wang, Qiang Zhao

**Affiliations:** 1 School of Basic Medical Sciences, Nanchang Medical College, Nanchang 330052, China; chenxianxin@ncmc.edu.cn; 2 State Key Laboratory of Food Science and Resources, Nanchang University, Nanchang 330047, China; 417900220158@email.ncu.edu.cn (X.Z.); 417900220094@email.ncu.edu.cn (S.W.); 3 College of Food and Health, Beijing Technology and Business University, Beijing 100048, China; ningfj@btbu.edu.cn

**Keywords:** rice dreg protein, transglutaminase, cross-linking, polymers, in vitro digestibility

## Abstract

The study examined how transglutaminase (TG)-induced cross-linking affects the structural, functional, and in vitro digestibility characteristics of rice dreg protein (RDP). Analysis using SDS-PAGE showed that low-molecular-weight fragments vanished, while high-molecular-weight polymers formed. Additionally, Fourier transform infrared (FTIR) spectroscopy demonstrated a reduction in β-sheet content alongside an elevation in β-turn structures as the cross-linking process became more pronounced, which was associated with a reduction in both total and free sulfhydryl groups. The hydrophobic nature of the surface and the emulsifying properties of cross-linked RDP initially rose but began to decrease when TG concentrations surpassed 10 U/g of protein. Conversely, emulsion stability and water-binding capacity decreased, while oil-binding capacity improved compared to native RDP. Solubility and in vitro digestibility decreased with cross-linking, whereas rheological properties significantly improved with higher TG levels. These findings suggest that controlled TG-mediated cross-linking (e.g., 10 U/g) effectively enhances the functional properties of RDP, making it a promising ingredient for applications in plant-based meats, baked goods, and fortified beverages within the food industry.

## 1. Introduction

The development of plant-based protein sources as alternatives to costly animal proteins has gained momentum due to their potential to meet the rising demands for techno-functional, tropho-functional, and sensory qualities in food [1]. Among these, rice dreg protein (RDP) is notable for its distinctive nutritional profile and hypoallergenic characteristics, which surpass those found in other proteins derived from cereals and legumes [2,3]. Rice dregs, the primary source of RDP, are a by-product generated during the wet-milling process of rice to produce rice starch or rice syrup. In this process, rice grains are steeped, ground, and separated into starch, protein, and fiber fractions, leaving behind a protein-rich residue—commonly referred to as rice dregs or rice protein by-product [4]. Comprising over 50% protein, RDP is abundant and cost-effective, particularly in southern China, where the rice starch syrup industry generates vast quantities of this resource [5]. However, its low digestibility and poor solubility limit its commercial value, with most RDP currently underutilized as animal feed—a practice that exacerbates environmental pollution through excessive nitrogen runoff [6].

To realize the full potential of RDP, researchers have investigated a range of modification techniques, which encompass physical, chemical, and enzymatic methods. For instance, enzymatic hydrolysis has improved the antioxidant activity and emulsion stability of RDP [7], while the Maillard reaction has enhanced its physicochemical properties and emulsion stabilization when conjugated with κ-carrageenan [5]. However, the Maillard reaction does have some disadvantages, which include unwanted browning, mutagenic compound formation and nutritional losses (one example is lysine). Hence, safer and better modifications are required [5].

Transglutaminase (TG) plays a crucial role in various biochemical processes. It specifically aids in the transfer of acyl groups between glutamine and lysine residues within proteins. This enzymatic activity leads to crosslink formation, which can occur either intra- or intermolecularly [8,9]. Cross-linking contributes to improved protein functionality via the formation of high-molecular-weight polymers, which enhance gelling, emulsifying and texture. Notably, RDP is primarily composed of glutelins and prolamins, which provide essential amino acids and contribute to its hypoallergenic nature and potential cholesterol-lowering effects [5,10]. The presence of reactive glutamine and lysine residues in these major protein fractions makes RDP a promising substrate for TG-mediated cross-linking.

TG has emerged as a highly efficient cross-linking enzyme with broad application potential in the modification of plant proteins. For instance, it has been proven to effectively improve the gelling, emulsifying, and foaming properties of gluten [11,12]. In cereal protein applications, TGase treatment not only significantly enhanced the elasticity and textural properties of brown rice batters [13] but also improved the in vitro digestibility of kidney bean phaseolin by increasing the cross-linking density [14]. Furthermore, remarkable results have been achieved using TGase to improve the gelation properties of soy protein isolate [15], enhance the foaming capacity of pea protein [16], and optimize the gelling properties of mung bean protein [17]. Recent research has expanded into mixed protein systems; for example, TGase has been used to concurrently modify rapeseed protein with wax-based oleogels to construct plant-based bigels with desirable textures [18] and has been applied in high-moisture extrusion to improve the fibrous structures of various plant proteins [19]. These systematic findings establish TGase as a key tool in developing innovative plant-based foods like meat analogs and dairy alternatives [20,21]. However, while the modifying effects and mechanisms of TGase on mainstream plant proteins (such as soy, pea, and wheat gluten) have been extensively studied, its application in rice-derived protein (RDP)—a by-product of rice processing—remains largely unexplored. Existing literature contains only limited studies on the preliminary effects of TGase on rice-based products [13]. A systematic investigation into how TGase-induced cross-linking affects the structural characteristics, functional properties (such as solubility and emulsifying stability), and nutritional quality (e.g., digestibility) of RDP is critically lacking. Concurrently, academic discussions concerning the optimal reaction conditions for TGase (e.g., concentration, temperature, pH) [22], as well as theoretical debates on the protein network formation mechanisms it induces [23], represent a complete knowledge gap within the context of RDP systems. This research lag is strikingly mismatched with the significant potential of RDP as a hypoallergenic and highly biocompatible protein source. 

Therefore, a systematic investigation into the effects of TGase modification on rice-derived protein—elucidating the interrelationships between cross-linking conditions, structural alterations, functional responses, and changes in nutritional properties—is imperative. Such research would not only fill a critical gap in the current landscape of enzymatic modification studies but also provide a novel theoretical foundation and technical pathway for developing high-value, functional rice protein products.

This study investigates the impact of TG treatment on RDP’s structure, function, and nutrition. We applied TG at concentrations of 5, 10, 20, and 50 U/g protein—selected to span low to very high levels. Concentrations of 5–20 U/g represent common ranges for plant protein modification. The 50 U/g level was included to probe functional saturation; this choice is supported by precedents in protein studies [24]. We evaluated emulsifying activity, stability, water/oil retention, rheology, and in vitro digestibility to identify the optimal TG dosage. Our findings demonstrate that TG-induced cross-linking effectively transforms RDP into a multi-functional ingredient, overcoming key limitations and supporting its broader use in sustainable food systems.

## 2. Materials and Methods

### 2.1. Material

Rice dreg (RD) powder obtained from Jiangxi Hengtian Co., Ltd. (Jiangxi, China) containing 60% protein, 5.2% moisture, and 3.8% ash (on a dry weight basis) was used in this study. TG (1000 U/g) was from Yiming Biological Products Co., Ltd. (Taixing, China). Pepsin (1000 U/mL) and trypsin (100 U/mL) were purchased from Sigma-Aldrich. Co., Ltd. All other chemical reagents were of analytical quality.

### 2.2. Extraction and Cross-Linking of RDP

Defatted RD underwent preextraction treatment following Shahid et al. [10]. The extracted RDP was reconstituted in deionized water to achieve a protein concentration of 4% (*w*/*w*). TG was subsequently added at various enzyme-to-substrate ratios (0, 5, 10, 20, 50 U/g protein). Then, the mixture was incubated with shaking at 37 °C for 3 h. The reactions were stopped by placing the samples quickly into an ice-water bath (4 °C, 30 min) before lyophilization for further analysis [25].

### 2.3. Sodium Dodecyl Sulfate-Polyacrylamide Gel Electrophoresis (SDS-PAGE)

The molecular weight distribution of native and TG-modified RDP was analyzed using SDS-PAGE on a Bio-Rad Mini PROTEAN^®^ 3 System (Bio-Rad Laboratories, Hercules, CA, USA). This method was based on the protocol established by Laemmli (1970) with slight adjustments [7], including the use of 12% and 3% acrylamide gels for separation and stacking, respectively. Before loading onto the gel, all samples were denatured in boiling water for 10 min. The protein bands were visualized using Coomassie Brilliant Blue R-250, after which they were destained in methanol/acetic acid/water (1:1:8, *v*/*v*/*v*).

### 2.4. Structural Characteristics of Cross-Linked RDP

#### 2.4.1. Fourier Transform Infrared Spectroscopy (FTIR) Analysis

The structural changes in RDP following TG modification were analyzed using FTIR. This method provides valuable insights into alterations to secondary structures (α-helices, β-sheets and random coils), as well as modifications to functional groups induced by enzymatic cross-linking. A Nicolet 5700 FTIR spectrometer (Thermo Nicolet Corporation, Waltham, MA, USA) was used within the wavenumber range of 400–4000 cm^−1^, with a resolution of 4 cm^−1^ and 32 scans accumulated.

#### 2.4.2. Intrinsic Emission Fluorescence Spectroscopy

Fluorescence spectra of the samples’ intrinsic emission were obtained utilizing a Hitachi F-4500 fluorophotometer (Hitachi Co., Kyoto, Japan). The samples were excited at a wavelength of 280 nm. The emission spectrum was recorded over a range of wavelengths from 290 to 420 nm, with a consistent gap width of 5 nm maintained throughout the measurement process.

#### 2.4.3. Determination of Sulfhydryl Groups

The sulphydryl group content of RDPs was assessed with Ellman’s reagent (DTNB), in accordance with the procedure outlined by Shi et al. [2]. Specifically, 30 mg of protein samples were dissolved in 10.0 mL of Tris-glycine buffer. To determine total sulfhydryl levels, the buffer contained 8 M urea. By contrast, the buffer was free of 8 M urea for measuring exposed sulfhydryl levels. Following that, 50 μL of Ellman’s reagent was incorporated into the mixtures and the mixtures were incubated in the dark at room temperature for one hour while being shaken continuously. After the incubation period, the absorbance of the solution was recorded at 500 nm, with the reagent buffer used as a control blank for comparison.

### 2.5. Determination of Functional Properties

#### 2.5.1. Determination of Solubility

The solubility of the protein was assessed using the technique established by Chen et al. [26]. Samples were mixed with deionized water at a concentration of 1% (*w*/*v*). The pH levels of these mixtures were adjusted to fall within the range of 3 to 10. Following this, the protein suspensions underwent magnetic stirring at ambient temperature for 30 min. After this stirring period, the mixture was subjected to centrifugation at 4800 rpm for 10 min. The protein concentration present in the supernatant was evaluated using Lowry’s method. A standard curve was created using bovine serum albumin.

#### 2.5.2. Determination of Emulsifying Activity and Emulsion Stability

The emulsifying activity index (EAI) and the emulsion stability index (ESI) for the sample were determined through a modified turbidimetric technique as outlined by Hu and Li [27]. Initially, 16 mL portions of 0.1% protein solutions, dispersed in a 10 mM phosphate buffer (pH 7.0), were blended with 4 mL of soybean oil. This process was performed using a T18 digital ULTRA-TURRAX homogenizer (IKA, Staufenberg, Germany) at 12000 rpm for one minute at ambient temperature to create an emulsion. Right after the homogenization process (at 0 min) and again after a duration of 10 min, 50 μL of the emulsion was meticulously pipetted 0.5 cm above the bottom of the container and subsequently dispersed into 5 mL of a 0.1% SDS (*w*/*v*) solution for analysis. The absorbance of the prepared solution was recorded at a wavelength of 500 nm. A 0.1% SDS solution served as the control. The protein concentration (g/ml) prior to the emulsification process is denoted as C. The oil volume fraction of the emulsion, represented by ϕ, is quantified as a volume/volume ratio (*v*/*v*), with a specific value of ϕ = 0.20. The absorbance measurements at 0 min (*A*_0_) and 10 min (*A*_10_) were employed to compute the EAI and ESI as below:

(1) $$\mathrm{EAI}(m^{2}/g)=\frac{2\times 2.303}{C\times (1-\phi )\times 10^{4}}\times A_{0}\times dilution$$

(2) $$\mathrm{ESI}(\%)=\frac{A_{10}\times 100}{A_{0}}$$

#### 2.5.3. Water/Oil Holding Capacity (WHC/OHC)

Water and oil holding capacity were measured by a modified method of Li et al. [28].

A sample of 0.5 g was combined with 20.0 mL of distilled water or refined soybean oil within a centrifuge tube (25 mL). Following this, the mixture was centrifuged at 4800 rpm for 15 min. The increases in weight per unit of the sample were noted and represented the corresponding water/oil holding capacity (g/g).

#### 2.5.4. Determination of Surface Hydrophobicity

The hydrophobicity of the RDP surface was assessed utilizing the fluorescent probe 1-anilino-8-naphthalene sulfonic acid (ANS). Initially, an RDP solution (0.05%, *w*/*v*) was prepared in a phosphate buffer (pH 7.0). To achieve uniform dispersion, this solution underwent homogenization for one minute at 12,000 g using a disperser. Following this, the homogenized mixture was gradually diluted with phosphate buffer to achieve concentrations between 0.005% and 0.025% (*w*/*v*).

For each concentration, 4 mL of the diluted sample was precisely combined with 20 μL of an ANS solution (8 mmol/L). Immediately after mixing, the samples were vortexed for 5 seconds to achieve thorough blending. The mixtures were then incubated in the dark for 10 min to avoid any interference from ambient light sources, which could affect the fluorescence measurement. After incubation, the intensity of fluorescence emission was assessed. This measurement was conducted using an excitation wavelength set at 390 nm, while the emission wavelength was recorded at 470 nm.

The hydrophobicity index of the surface was assessed through the execution of a linear regression analysis. The process involved plotting the fluorescence intensity in relation to the concentration of RDP. The resulting graph was analyzed to determine the slope of the line, which served as the surface hydrophobicity index. This approach provides a quantitative evaluation of the hydrophobic characteristics of the RDP surface.

### 2.6. Determination of Rheological Properties

#### 2.6.1. Apparent Viscosity

The viscosity that appears in the protein dispersion created in water (12%, *w*/*v*) was assessed utilizing a stress-controlled rheometer (DHR-2, TA Instruments, New Castle, DE, USA). For the measurements, a plate-plate geometry (40 mm diameter, 1 mm gap) was employed. The samples were placed on the rheometer and permitted to stabilize at 25 °C for a duration of 5 min. The apparent viscosity of the sample dispersions was recorded across various shear rates, which ranged from 0.1 to 100 s^−1^.

#### 2.6.2. Dynamic Oscillation Rheology

Gelation profiles of protein sample suspensions (16%, *w*/*v*) during heating were investigated using dynamic oscillatory rheometry (DHR-2, TA Instruments, USA) with a parallel plate system (40 mm diameter, 1 mm gap). To prevent dehydration, a thin layer of silicone oil was applied, and the plate was insulated to reduce heat loss.

The samples were incubated at 50 °C for 30 min after loading, then heated to 95 °C at a rate of 5 °C/min and held for 10 min. They were subsequently cooled to 25 °C at the same rate and maintained at that temperature for 5 min. Measurements were taken at a frequency of 1 Hz and a strain of 0.1%, within the linear viscoelastic range. Before sample removal, a frequency sweep was conducted at 25 °C, covering 0.01 to 10 Hz with a strain of 1%.

### 2.7. Digestibility In Vitro

The in vitro evaluation of protein sample digestibility was conducted using the methodologies described by Wang et al. [8], which simulates the gastric and intestinal phases. To prepare 1% (*w*/*v*) protein suspensions, the protein samples were initially dispersed in a phosphate buffer (pH 8.0). Following this, the solution pH was adjusted to 2.0 using 1 M HCl to simulate the gastric phase and porcine pepsin (2%, *w*/*v*, P-7000, Sigma-Aldrich, Burlington, MA, USA) was included. The hydrolysis process was conducted at 37 °C for periods spanning from 0 to 120 min. To stop the hydrolytic process, an equivalent volume of 20% (*w*/*v*) trichloroacetic acid (TCA) was introduced. The resulting suspension underwent heat treatment at 90 °C for 5 min, after which it was cooled to 4 °C for subsequent analysis.

In the two-step hydrolysis protocol, the initial reaction mixture was prepared and subjected to pepsin hydrolysis according to the standardized procedure. Once the pepsin digestion was complete, the enzymatic reaction was terminated through heat application at 90 °C for 5 min, followed by rapid cooling to 4 °C. Subsequently, the sample underwent lyophilization to facilitate subsequent enzymatic processing. The lyophilized samples underwent a regeneration process in a solution consisting of 10 mL of 0.2 M phosphate buffer, adjusted to a pH of 8.0 to simulate the intestinal phase. To facilitate the digestion of the samples, 5% (*w*/*v*) trypsin (T-7409, Sigma-Aldrich, Burlington, MA, USA) was introduced into the mixture. The digestive reaction was then incubated at 37 °C for a period varying from 0 to 120 min. Following the incubation, the resulting protein precipitates were isolated through centrifugation at 5000 rpm for 20 min. Ultimately, the quantity of nitrogen that was soluble in TCA and released during the enzymatic digestion was determined by assessing the absorbance at 280 nm in the supernatant fractions. These quantitative data were utilized to evaluate and compare the in vitro digestibility profiles.

### 2.8. Statistical Analysis

All measurements were performed in triplicate (*n* = 3) and repeated twice (independent experimental runs), with results expressed as means ± standard deviations. ANOVA was performed using Origin Pro 8.0, and statistical significance was set at *p* < 0.05 via the Tukey test.

## 3. Results and Discussion

### 3.1. SDS–PAGE Analysis

**Figure 1:**
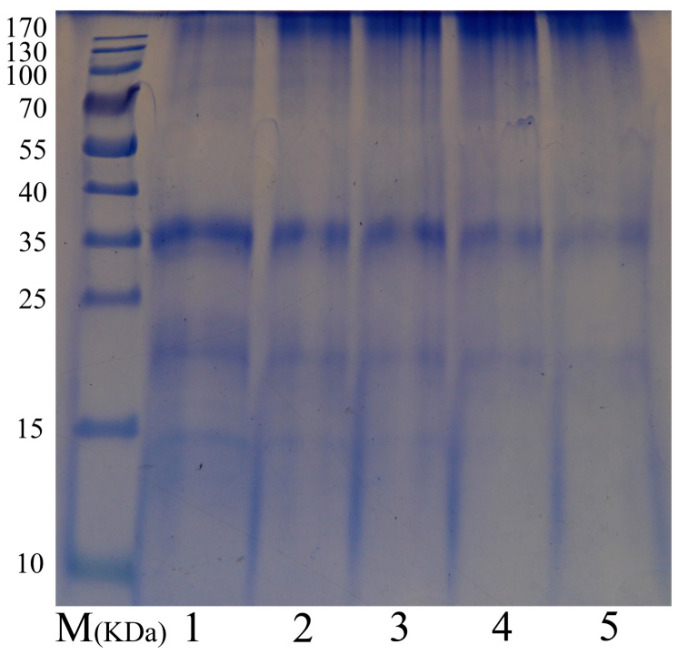
SDS-PAGE profile of native and TG-treated RDP with different enzyme concentrations. Lane M: Molecular weight marker; Lane 1: Native RDP (0 U/g); Lanes 2–5: TG-treated RDP at 5, 10, 20, and 50 U/g protein, respectively.

Electrophoresis analysis was conducted on native RDP, cross-linked RDP, and a protein molecular weight marker (10–170 kDa) to characterize protein band molecular weights and size profiles, as shown in Figure 1. Native RDP (lane 1) displayed three major subunits with molecular weights (Mw) near 35, 20, and 15 kDa, consistent with prior findings by Zhao et al. [7]. These bands are tentatively assigned as putative glutelins and albumins based on their approximate molecular weights. In samples modified by TG (lanes 2–5), a progressive decrease in the intensity of these three subunits was observed as the concentration of TG increased. Meanwhile, new peptide bands with high molecular weights (≥170 kDa) emerged at the upper part of the gel. The intensity of the high-molecular-weight bands observed in the study is directly proportional to the TG dosage, which indicates that TG induces the cross-linking of RDP subunits to form polymeric structures.

The disappearance of low-Mw subunits (15–35 kDa) and the concurrent appearance of high-Mw polymers (≥170 kDa) in TG-treated RDP (Figure 1) provide direct evidence of intermolecular cross-linking. Based on the molecular weights of the diminishing bands, which correspond to the major glutelin and prolamin fractions in RDP [7], we can infer that these are the primary components participating in the cross-linking reaction. This is consistent with the known mechanism of TG, which catalyzes bonds between glutamine (abundant in glutelin) and lysine residues, and suggests that the structure of these RDP fractions makes them highly susceptible to TG-mediated polymerization.

Wu et al. [14] observed analogous reductions in peak intensities during TG-mediated cross-linking of rice bran protein (RBP), attributing this to the formation of polymerized proteins. In this research, the lack of discernible new polypeptides within the resolvable Mw range (below 170 kDa) suggests that TG-induced polymerization primarily yielded ultra-large aggregates exceeding the resolution limit of the gel. Collectively, SDS-PAGE analysis confirms the formation of high-Mw protein polymers in RDP via TG catalysis, consistent with the expected mechanism of transglutaminase-driven inter- and intra-molecular cross-linking.

### 3.2. Structural Characteristics of the Cross-Linked RDP

#### 3.2.1. FTIR Spectroscopy

**Figure 2:**
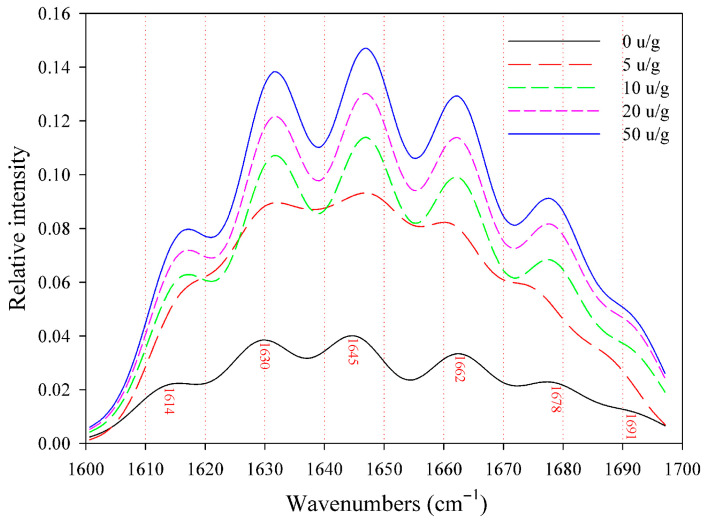
FTIR spectra (400–4000 cm^−1^) of native and TG-treated RDP at different enzyme concentrations (5–50 U/g). Progressive changes in amide I and II bands indicate alterations in secondary structure.

FTIR spectroscopy serves as an effective method for examining alterations in protein structure, especially in denatured or aggregated forms [29]. As shown in Figure 2, the FTIR spectra for cross-linked RDP displayed a qualitatively comparable amide band pattern to that of native RDP, which is consistent with the results reported by Song and Zhao [30] regarding soy protein and its cross-linked variant. Notwithstanding this spectral similarity, quantitative differences emerged with increasing TG concentrations: the relative intensity of amide bands increased progressively with TG level, and absorbance values showed a corresponding upward trend.

According to Zhao et al. [31], the differences in secondary structures between native and cross-linked RDP were examined through the deconvolution of the amide I band (1600–1700 cm^−1^). The quantitative analysis of secondary structure via FTIR amide I band deconvolution is a well-established method, as demonstrated in studies on various food proteins [32,33]. The amide I spectrum of RDP included six distinct absorbance peaks, which corresponded to β-sheet, β-turn, and random coil configurations. Notably, TG-induced cross-linking triggered a quantifiable shift in secondary structure composition: cross-linked RDP exhibited a decrease in β-sheet content and a concomitant increase in β-turn structures, with no detectable α-helix conformations—consistent with prior observations in RDP [7].

Quantitative analysis via curve-fitting peak area calculations (Table 1) revealed that TG-mediated cross-linking correlated with a reorganization of protein secondary structure, favoring β-turn formation over β-sheet structures. This change in structure probably indicates the creation of biopolymers with high molecular weight through the process of TG-catalyzed isopeptide bond formation, potentially leading to localized folding or conformational constraints within the protein matrix. In contrast to the results observed with peanut protein, the combination of microfluidization and TG treatment facilitated unfolding and disaggregation [34]. This highlights how the effects of modification techniques on protein structure can vary depending on the context. These changes in secondary structure significantly impact the functional properties of RDP: The reduction in β-sheet content and increase in β-turn structures may enhance the flexibility and interfacial activity of protein molecules, thereby improving their emulsifying properties [35].

Simultaneously, the more ordered β-turn structures contribute to the formation of a denser and more stable three-dimensional gel network, enhancing the water-holding capacity and mechanical strength of gels [36]. This provides a structural basis for the application of RDP in food systems.

These changes in secondary structure provide a structural basis for the observed functional properties. The reduction in β-sheet content, often associated with a more rigid and ordered structure, and the increase in more flexible β-turn structures, likely contribute to the increased flexibility and interfacial activity of the cross-linked proteins, thereby explaining the improved emulsifying properties described in Section 3.3.2. Furthermore, the increase in β-turn content is indicative of a structural reorganization where polypeptide chains realign and bend to accommodate the newly formed isopeptide cross-links. This restructured, densely packed, and less-hydrated matrix, as part of the high-molecular-weight aggregates, directly contributes to the observed decrease in protein solubility, as discussed in Section 3.3.1.

#### 3.2.2. Intrinsic Emission Fluorescence Spectra

**Figure 3:**
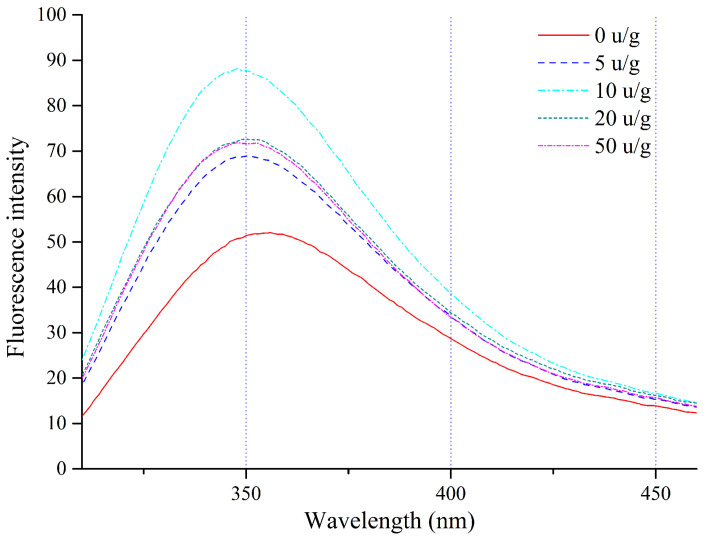
Intrinsic fluorescence spectra (excitation = 280 nm; emission = 290–420 nm) of native and TG-modified RDP.

The process of probing the unfolding or aggregation of RDP involves assessing the intrinsic fluorescence intensity at excitation wavelengths that correspond to residues of tryptophan (Trp) or tyrosine (Tyr), in addition to conducting analyses of surface hydrophobicity [37]. Fluorescence spectra of intrinsic emission from both native and cross-linked RDP dispersions (excitation at 280 nm) were recorded over 290–420 nm, as shown in Figure 3. In the case of cross-linked RDP, the highest fluorescence intensity (Iₘₐₓ) observed in the emission profile rose steadily as the TG concentration increased from 0 to 10 U/g, but it declined at higher TG levels. Concomitantly, A decrease in fluorescence intensity and λₘₐₓ shift collectively reflect protein unfolding or conformational changes, likely due to masking of chromophores (Trp, Tyr, Phe residues and surface-exposed -SH groups) by TGase-induced polymerization, which sequesters fluorescent moieties into internalized or cross-linked domains [38]; or partial deamidation of glutamine/asparagine residues at high TG concentrations, which increases electrostatic repulsion and disrupts polymer packing [12]. This biphasic pattern—initial intensity increases followed by decrease—suggests that TG induces concentration-dependent structural transitions in RDP, balancing polymerization-driven aggregation (enhanced fluorescence via reduced solvent exposure) and over-cross-linking-induced charge repulsion or chromophore burial.

#### 3.2.3. Sulfhydryl Groups (-SH) Contents

The total and free sulfhydryl (SH) levels in both untreated and cross-linked RDP were displayed (Table 2), with native RDP showing levels of 5.00 (total SH) and 3.33 (free SH) μmol/g protein, respectively. TG treatment induced a significant dose-dependent decrease in both total and exposed SH levels. The significant dose-dependent decrease in both total and free sulfhydryl groups upon TG treatment (Table 2) is a key chemical indicator of the cross-linking process. The consumption of free -SH groups can be attributed to their oxidation into new disulfide bonds (S-S) during the enzymatic reaction and incubation. Furthermore, the formation of ε-(γ-glutamyl)lysine isopeptide bonds, the primary cross-link catalyzed by TG, can lead to the burial and inaccessibility of -SH groups within the newly formed high-Mw polymer structures, thereby reducing the measured free -SH content, as previously reported in soy protein [25] and casein [39]. This convergence of evidence from SDS-PAGE and sulfhydryl analysis strongly supports the occurrence of TG-mediated chemical cross-linking in RDP.

Collectively, FTIR spectroscopy, sulfhydryl group quantification, and intrinsic fluorescence analyses provide converging evidence that TG treatment induces structural remodeling of RDP, including secondary structure reorganization, chromophore masking, and disulfide bond-mediated aggregation. These findings establish TG-catalyzed cross-linking as a viable strategy to modulate RDP’s macromolecular architecture for improved functional properties in food applications.

### 3.3. Protein Functional Properties

#### 3.3.1. Solubility

**Figure 4:**
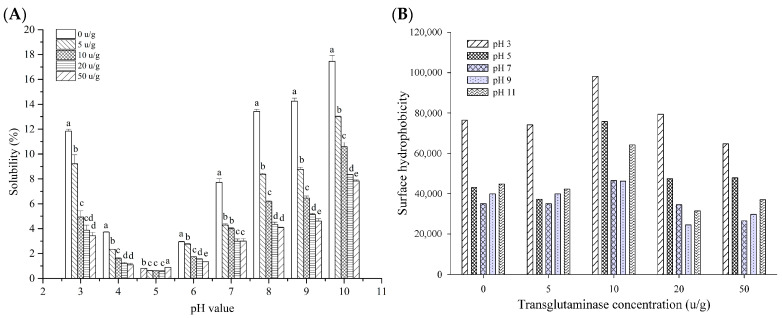
(**A**) Effect of pH (3–10) on the solubility (%) of native and TG-treated RDP (1% *w*/*v*, 25 °C). (**B**) Surface hydrophobicity (H_0_) determined using ANS probe across pH 3–11. Error bars represent standard deviations (*n* = 3). Different letters indicate statistically significant differences among sample means (*p* < 0.05, Tukey’s HSD test). Samples sharing the same letter at a given point are not significantly different.

The solubility of food proteins is an essential functional characteristic that significantly affects their emulsifying, foaming, and rheological properties [40]. An assessment of the solubility of both native and cross-linked RDP was conducted over a pH spectrum of 3 to 10 (Figure 4A). All samples exhibited a U-shaped solubility profile: solubility decreased sharply at pH 5 (isoelectric point) and gradually increased at pH > 5 or <5, with the lowest solubility occurring at pH 5, consistent with the charge-driven aggregation behavior of proteins near their pI.

Significantly, the solubility of cross-linked RDP exhibited a decrease that depended on the dosage as the concentration of TG increased, especially under highly acidic (pH 3) and alkaline (pH > 7) environments. This is due to TG-induced cross-linking of proteins, which facilitates the development of elongated polypeptide chains and high-molecular-mass aggregates. These structural changes limit protein hydration and dispersibility, a mechanism previously observed in vicilin-rich kidney bean proteins [41] and TG-treated peanut proteins.

This decrease in solubility is a direct consequence of the structural changes induced by cross-linking. Specifically, the formation of high-molecular-weight aggregates (as confirmed by SDS-PAGE) and the marked shift in secondary structure towards more β-turns (as revealed by FTIR) are key factors. The rise in β-turns reflects a molecular rearrangement that supports the formation of a densely packed, cross-linked network. Within this network, the availability of polar groups for water interaction is reduced, creating a less hydratable protein matrix. These changes collectively diminish the protein’s hydration capacity and dispersibility. In contrast to pH-shifting methods that enhance solubility in soy or faba bean proteins (e.g., 7S/11S globulins) [7], the divergent behavior in RDP likely stems from intrinsic differences in protein composition and subunit structure. RDP’s gluten-like fractions and dominant low-molecular-weight subunits may render them more susceptible to TG-mediated aggregation, whereas legume proteins with higher globulin content respond differently to pH or cross-linking treatments.

#### 3.3.2. Emulsifying Activity and Emulsion Stability

The emulsifying properties and stability of protein emulsions are affected by factors such as molar mass, conformational integrity, hydrophobic characteristics, surface charge, and various physicochemical conditions (for instance, pH, ionic strength, and temperature) [42]. The EAI and ESI of cross-linked RDP are shown in Table 2. With increasing TG concentration, the EAI first increased and then plateaued at TG ≥ 10 U/g, aligning with prior reports on TG-improved emulsifying activity in black bean protein isolate [43]. In that study, the EAI peaked at 20 U/g TG, whereas in casein, TGase caused an impaired EAI [39].

The EAI enhancement in RDP is attributed to TG-induced cross-linking, which exposes polar and hydrophobic groups on the protein surface, promoting oil-phase binding. Concurrently, hydrophobic interactions drive protein aggregation at the oil-water.

For the ESI, TG cross-linking significantly improved emulsion stability, likely due to increased negative charge density from blocked lysine residues, which sterically stabilizes droplets. Analogous ESI enhancements via charge modulation have been observed in ovalbumin [44] and gelatin [45]. These findings collectively demonstrate that TG treatment can tailor RDP’s amphiphilic properties to enhance emulsification, with optimal performance achieved at moderate TG concentrations.

The enhancement in emulsifying properties can be mechanistically interpreted by the structural modifications induced by TG cross-linking. 

Emulsifying Activity Index (EAI): The initial improvement is attributed to the partial unfolding and reorganization of protein molecules, which increases the exposure of both hydrophobic and hydrophilic residues. This enhanced amphiphilicity allows the cross-linked proteins to act as more effective surfactants, rapidly adsorbing at the oil-water interface during homogenization to reduce interfacial tension. The formation of soluble protein polymers may also provide a larger molecular footprint at the interface, contributing to the formation of smaller oil droplets and thus a larger interfacial area (higher EAI). However, at excessively high TG levels (e.g., 50 U/g), over-aggregation can occur, leading to larger, insoluble polymers that diffuse and rearrange more slowly at the interface, resulting in the observed plateau or slight decline in the EAI. 

Emulsion Stability Index (ESI): The remarkable improvement in stability is primarily due to the formation of a viscoelastic, cross-linked protein layer at the oil droplet interface. This robust interfacial membrane provides a strong mechanical barrier against droplet coalescence. Furthermore, the cross-linking reaction consumes lysine residues, which may increase the net negative charge on the protein surface, thereby enhancing electrostatic repulsion between adjacent oil droplets. The formation of a three-dimensional network in the continuous aqueous phase, as suggested by the improved rheological properties (Section 3.4), further acts as a physical barrier, hindering droplet movement and creaming, thereby significantly improving long-term stability.

#### 3.3.3. Water/Oil Holding Capacity

The ability of proteins to retain water is essential for minimizing moisture loss and preserving the freshness of baked goods. According to Table 2, the WHC of RDP was measured at 5.16 g/g, surpassing that of cross-linked RDP, which had values of 4.362, 4.184, 4.360, and 4.505 g/g. Alabi et al. [37] indicated that the values for WHC in viscous foods varied between 1.49 and 4.72 g/g. Consequently, cross-linked RDP fulfills this criterion and is suitable for use in products that require significant water retention. Conversely, it has been demonstrated that TG-induced cross-linking improves the WHC of pea protein [46] as well as soy-hemp-wheat protein meat substitutes [47]. This effect may be linked to the reduced solubility of cross-linked RDP and the creation of high-molecular-weight biopolymers.

Cross-linked RDP showed a higher OHC (5.15, 6.82, 7.18, 7.09 g/g) than native RDP (4.30 g/g, Table 2). The OHC of flours from wheat, barley, and soybean was also elevated by TG-induced cross-linking. This increased capacity in TG-treated RDP could be attributed to the cross-linking process and the greater exposure of non-polar side chains from hydrophobic groups within the RDP molecules. However, at a TG concentration of 50 U/g, the OHC slightly decreased, which could be attributed to deamination by TG, converting amide bonds to -COOH groups and increasing polarity [12]. The high OHC of modified RDP offers potential applications in retaining flavor, improving palatability, and extending shelf life.

The divergent trends in WHC and OHC can be mechanistically explained by the distinct nature of water-protein and oil-protein interactions, both of which are profoundly influenced by TG-induced cross-linking.

Mechanism for Decreased Water-Holding Capacity (WHC): The observed reduction in WHC for cross-linked RDP is primarily attributed to the decrease in protein solubility and the formation of large, densely packed aggregates. Cross-linking reduces the number of individual protein molecules and their associated hydrophilic groups (e.g., -OH, -NH_2_, -COOH) available to form hydrogen bonds with water. Furthermore, the tight, cross-linked network structure offers less space for physical entrapment and capillary action to retain water molecules within the protein matrix, leading to easier water expulsion during centrifugation. This phenomenon has been consistently reported in other cross-linked plant proteins.

Mechanism for Increased Oil-Holding Capacity (OHC): Conversely, the significant enhancement in OHC is driven by the increased exposure of hydrophobic regions and the creation of a robust physical network. TG-mediated cross-linking often induces partial unfolding or structural rearrangement of protein molecules, which can expose previously buried non-polar amino acid side chains. These exposed hydrophobic sites can form strong hydrophobic associations with the oil phase. More importantly, the cross-linked, high-molecular-weight polymers form a coarse, porous, and rigid gel-like structure with a larger surface area and stronger mechanical integrity. This structure is highly effective at physically entrapping and immobilizing oil droplets through capillary forces, preventing oil release under centrifugal force. The slight decrease in OHC at the highest TG concentration (50 U/g) might be due to excessive cross-linking leading to a more brittle network with reduced porosity or partial deamination increasing surface polarity, thereby weakening hydrophobic interactions.

#### 3.3.4. Surface Hydrophobicity

The investigation into the surface hydrophobicity of modified RDP was conducted across a pH spectrum ranging from 3 to 11, as illustrated in Figure 4B. The findings demonstrated a consistent pattern: as the pH rose, the surface hydrophobicity of the samples initially decreased before subsequently increasing. The observed pH-dependent variations in surface hydrophobicity originate from dynamic structural reorganizations of protein molecules under different pH conditions. Initially, increasing pH induces molecular compaction through increased electrostatic repulsion between charged residues, leading to structural contraction. This conformational compactness reduces solvent exposure of internal hydrophobic moieties, thereby decreasing surface hydrophobicity. However, as pH continues to rise beyond the physiological optimum, alkaline-induced denaturation occurs via partial unfolding of tertiary structures or interchain aggregation. These processes disrupt the native packing arrangement, resulting in re-exposure of previously buried hydrophobic domains and subsequent restoration of surface hydrophobicity.

As shown in Figure 4B, surface hydrophobicity values of cross-linked RDP decreased with increasing cross-linking extent from 0 to 10 U/g and then increased. This result aligns with the pattern observed in intrinsic emission fluorescence spectra. It is hypothesized that TG-induced cross-linking leads to protein aggregation via hydrophobic and electrostatic forces, thereby preventing hydrophilic groups from ANS binding. Notably, TG-induced cross-linking of soybean protein resulted in higher surface hydrophobicity [30,48], whereas acacia gum conjugation of soybean protein or glucose conjugation of β-lactoglobulin caused lower surface hydrophobicity. Furthermore, the oligomerization process of milk proteins has been demonstrated to reveal protein structures, bringing to light hydrophobic amino acid residues that are hidden within the tertiary configuration of native protein molecules [49]. This phenomenon plays a role in enhancing the surface hydrophobicity of cross-linked proteins.

### 3.4. Rheological Properties

**Figure 5:**
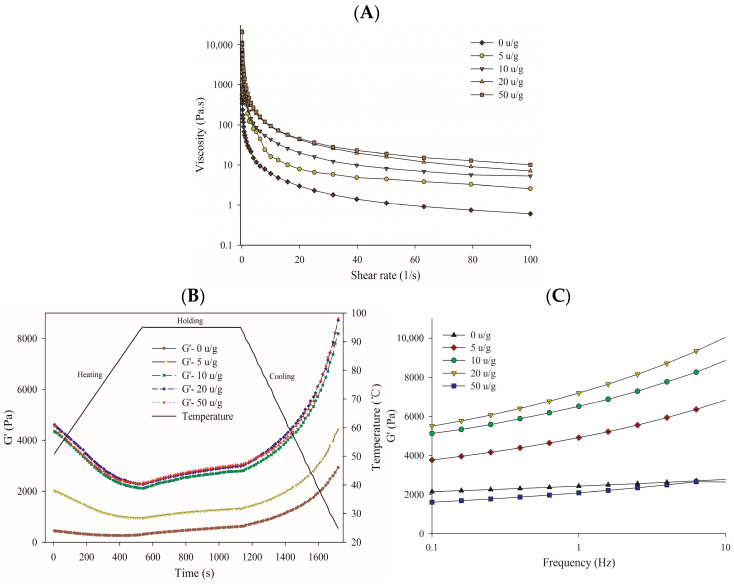
(**A**) Apparent viscosity (Pa·s) as a function of shear rate for native and TG-cross-linked RDP (12% *w*/*v*, 25 °C). (**B**) Evolution of storage (G′) during heating–cooling cycle (1 Hz, 0.1% strain). (**C**) Frequency sweeps (0.01–10 Hz, 1% strain) of gels formed at 25 °C.

The flow characteristics of the dispersions (16% *w*/*v*) made with RDP and cross-linked RDP are illustrated in Figure 5A. Each of the dispersions demonstrated thixotropic properties (shear thinning), and a notable rise in apparent viscosity was noted for the TG-catalyzed cross-linked RDP. This result is consistent with previous findings on cross-linked casein micelles [50], soybean protein [25], and casein [39] treated with TG. The rheological properties of a protein suspension are influenced by factors such as molecular size, shape, interactions with ionic solvents, conformation, hydrodynamic volume, and flexibility in the hydrated state [51]. In this study, the development of protein polymers characterized by greater molecular weights and larger molecular volumes primarily contributed to the rise in apparent viscosity.

The protein concentration for rheological testing (12% for apparent viscosity, 16% for dynamic oscillatory tests) was selected to be representative of concentrated food systems. When a protein solution is heated, it results in the unfolding of molecules, which subsequently causes aggregation. The gelling properties of RDP and cross-linked RDP were examined throughout a heating-holding-cooling cycle (Figure 5B). The protein suspensions were raised in temperature from 25 to 95 °C, maintained at 95 °C for a duration of 10 min, and then cooled back down to 25 °C. During this process, the storage modulus (G’) was measured as a function of the heating duration.

The evolution of the storage modulus (G′) was monitored as a key indicator of elastic network formation. During the initial heating stage, both native and cross-linked RDP exhibited a sharp drop in G′, attributable to protein denaturation and the disruption of weak interactions. Throughout the holding period at 95 °C, rearrangement of the protein network and the formation of new covalent (disulfide) and non-covalent bonds occurred. A significant rise in G′ was observed during the subsequent cooling phase, as the temperature decreased from 95 °C to 25 °C. This marked increase in elasticity is a hallmark of heat-induced gelation, driven by the reassociation of unfolded protein chains and the exposure of hydrophobic groups, leading to the formation of a three-dimensional network [52,53].

The cross-linked RDP exhibited notably higher G′ values than the native RDP across the entire thermal profile (Figure 5B). This demonstrates that the TG treatment led to the formation of a stronger, more elastic gel network, consistent with the formation of larger, denser protein aggregates via cross-linking.

The mechanical spectra of the protein samples were analyzed using a frequency sweep at a steady strain of 1%, a temperature of 25 °C, and an oscillation frequency ranging from 0.01 to 10 Hz, as shown in Figure 5C. The G′ values for all protein samples showed little variation across the entire frequency spectrum (0.01 to 10 Hz), which is characteristic of a covalently cross-linked gel structure with weak frequency dependence [52]. This behavior indicates a stable, solid-like network. TG-induced cross-linked RDP exhibited significantly higher G′ values than native RDP, confirming the enhancement of gel strength through enzymatic cross-linking. However, at a TG concentration of 50 U/g, the G′ value decreased, which could be attributed to excessive cross-linking leading to a more brittle or heterogeneous network structure, or partial deamination affecting protein-solvent interactions [54].

Collectively, the rheological data allow us to infer the structural characteristics of the formed gels. The higher G′ values and apparent viscosity of cross-linked RDP, particularly at moderate TG levels (10–20 U/g), indicate the formation of a denser and more cross-linked gel network compared to the weaker network of native RDP. This enhanced structural integrity is likely due to TG-induced ε-(γ-glutamyl)lysine isopeptide bonds acting as additional covalent cross-links within the protein matrix, reinforcing the network. This result aligns with findings on TG-treated soy protein [25] and pea protein [46], where cross-linking also led to stronger gels. However, the unique subunit composition of RDP (rich in glutelin and prolamin) appears to make it particularly responsive to TG modification, resulting in a pronounced improvement in rheological properties that may surpass that of some legume proteins.

### 3.5. In Vitro Digestibility

**Figure 6:**
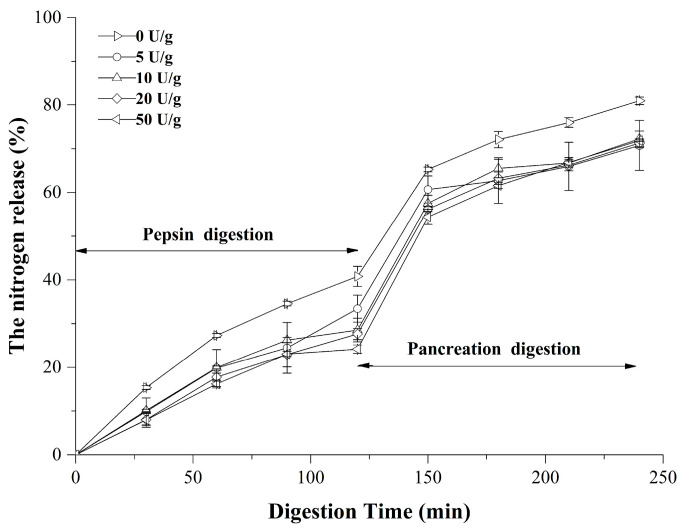
In vitro digestibility of native and TG-modified RDP determined by TCA-soluble nitrogen release during sequential pepsin (0–120 min) and trypsin (0–120 min) digestion at 37 °C. Data are mean ± SD (*n* = 3).

Digestibility serves as a key indicator of protein bioavailability [55]. The assessment of the in vitro digestibility for both native and cross-linked RDP was conducted, with the findings illustrated in Figure 6**.** All protein samples exhibited comparable digestion trends: the amount of TCA-soluble nitrogen dropped with the degree of cross-linking. During the initial 120 min of pepsin digestion, followed by a further 120 min of trypsin digestion, TCA-soluble nitrogen increased rapidly and then slowed down. Notably, cross-linked RDP exhibited significantly lower TCA-soluble nitrogen than native RDP throughout the in vitro digestion process. The digestion pattern of RDP was analogous to that found by Jiang and Zhao [25], who demonstrated that TGase-induced cross-linking of soy protein decreased pepsin + trypsin digestibility compared to its native counterpart.

Proteins that are highly cross-linked can create steric hindrance, preventing proteolytic enzymes from reaching peptide bonds. This results in a decrease in enzymatic digestibility in vitro [56].The structural features of proteins and the substrate specificity of enzymes can limit hydrolysis by pepsin and trypsin. Earlier research has indicated that enhanced enzyme digestibility is positively associated with the degree of cross-linking [41]. From a mechanistic perspective, enzymatic cross-linking balances two opposing factors to affect protein digestibility: protein unfolding/denaturation (which has a positive effect) and protein aggregation/polymerization (which has a negative effect) [41]. It is conceivable that the structural modifications in cross-linked RDP have a net negative impact on in vitro digestibility. The property of TG treatment reducing the in vitro digestibility of rice residue protein is primarily utilized in the food industry to develop special dietary foods that require slow amino acid release and sustained energy supply. This characteristic makes it suitable for weight management products (by delaying gastric emptying and providing prolonged satiety), foods designed for diabetics (to avoid sharp postprandial blood glucose fluctuations), and sports nutrition products (to supply athletes with sustained energy and amino acids). Additionally, the reduction in digestibility is sometimes accompanied by a decrease in protein allergenicity, which may contribute to the development of low-allergen-risk foods. The technical principle lies in the fact that enzymatic treatment may promote cross-linking of protein molecules or their binding with other components in rice residue (such as phenolic compounds), masking the cleavage sites of proteases and thereby slowing down the digestion process.

## 4. Conclusions

This study systematically examined the structural and functional modifications of RDP through TG-induced cross-linking, offering valuable insights for its applications in food. The key findings indicated that TG-mediated cross-linking significantly altered RDP’s molecular structure, which confirmed the formation of high-molecular-weight polymers and structural shifts from β-sheets to β-turns. These structural changes had a direct impact on the functional properties: emulsifying activity peaked at 10 U/g TG, while excessive cross-linking (>10 U/g) reduced surface hydrophobicity and emulsification stability. Additionally, cross-linked RDP displayed enhanced rheological properties, including increased apparent viscosity and improved gel network formation during thermal processing, making it suitable for use in texture-modified food. Furthermore, TG treatment significantly reduces the in vitro digestibility of proteins, and this low digestibility enables the protein to delay its decomposition and absorption processes in the gastrointestinal tract, thereby endowing food with a sustained-release function and prolonging the release time of nutrients. Optimal TG concentration (10 U/g) emerged as a practical threshold for maximizing emulsifying capacity without compromising solubility or emulsion stability. These findings position TG-modified RDP as a promising ingredient for hypoallergenic, cholesterol-lowering functional foods. The enhanced emulsifying capacity and stability suggest its suitability for applications such as mayonnaise, creamy sauces, and salad dressings. The significantly improved rheological properties, including increased viscosity and gel strength, make it ideal for use in texture-modified foods, plant-based meat analogs to improve bite and juiciness, baked goods to enhance water retention and softness, and fortified beverages to increase protein content without compromising mouthfeel. However, future work should address limitations, including long-term stability under industrial processing conditions and in vivo validation of digestibility outcomes. Overall, this research advances sustainable valorization of rice byproducts, aligning with global efforts to transform low-value agro-residues into high-quality protein sources for food applications.

## Figures and Tables

**Table 1:** Estimation of the secondary structure from FTIR measurement.

Samples	β-Sheet (%)	β-Turn (%)	Random Coil (%)
Without TG	49.53	24.78	23.40
+TG, 5 U/g	49.34	25.16	23.30
+TG, 10 U/g	48.60	25.84	23.63
+TG, 20 U/g	48.58	26.22	23.33
+TG, 50 U/g	48.56	25.26	23.46

**Table 2:** Determination of the Sulfhydryl Group Content and Functional Properties of native and cross-linked RDP.

Index	Without TG	+TG, 5 U/g	+TG, 10 U/g	+TG, 20 U/g	+TG, 50 U/g
Total-SH (μmol/g)	5.00 ± 0.04 ^a^	1.12 ± 0.01 ^c^	1.26 ± 0.01 ^b^	1.26 ± 0.01 ^b^	1.22 ± 0.04 ^b^
Free-SH (μmol/g)	3.33 ± 0.01 ^a^	0.80 ± 0.03 ^b^	0.57 ± 0.03 ^d^	0.46 ± 0.01 ^e^	0.67 ± 0.02 ^c^
EAI (m^2^/g)	5.91 ± 0.03 ^a^	8.07 ± 0.04 ^b^	10.72 ± 0.06 ^c^	10.46 ± 0.06 ^d^	10.09 ± 0.04 ^e^
ESI (%)	76.0 ± 1.5 ^a^	96.15 ± 3.15 ^b^	98.04 ± 0.08 ^bc^	99.89 ± 0.42 ^c^	99.89 ± 0.48 ^c^
WHC (g/g)	5.16 ± 0.09 ^a^	4.36 ± 0.04 ^b^	4.18 ± 0.68 ^c^	4.36 ± 0.12 ^d^	4.51 ± 0.16 ^d^
OHC (g/g)	4.30 ± 0.07 ^a^	5.15 ± 0.15 ^b^	6.82 ± 0.15 ^c^	7.177 ± 0.15 ^d^	7.09 ± 0.18 ^d^

Average values ± standard deviations (*n* = 3). Different letters indicate statistically significant differences among sample means (*p* < 0.05, Tukey’s HSD test). Samples sharing the same letter at a given point are not significantly different.

## Data Availability

The original contributions presented in this study are included in the article Further inquiries can be directed to the corresponding author.

## References

[1] Olatunde O.O., Owolabi I.O., Fadairo O.S., Ghosal A., Coker O.J., Soladoye O.P., Aluko R.E., Bandara N. (2023). Enzymatic Modification of Plant Proteins for Improved Functional and Bioactive Properties. Food Bioprocess Technol..

[2] Shi Q., Lu W., Wang R., Hu J., Zhu J., Zhang H., Zhou N., Xiong Q. (2024). Lipidomic Analysis of Grain Quality Variation in High Quality Aromatic Japonica Rice. Food Chem. X.

[3] Shu W., Shi W., Xie H., Wang S., Zhang Q., Ouyang K., Xiao F., Zhao Q. (2025). Non-Covalent Interaction of Rice Protein and Polyphenols: The Effects on Their Emulsions. Food Chem..

[4] Luo J., Wang S., Xie H., Ouyang K., Wang C., Yang Y., Xiong H., Zhao Q. (2024). Improving the Functional Properties of Rice Glutelin: Impact of Succinic Anhydride Modification. Int. J. Food Sci. Technol..

[5] Du Y., Shi S., Jiang Y., Xiong H., Woo M.W., Zhao Q., Bai C., Zhou Q., Sun W. (2013). Physicochemical Properties and Emulsion Stabilization of Rice Dreg Glutelin Conjugated with κ-Carrageenan through Maillard Reaction. J. Sci. Food Agric..

[6] Pan L., Chen F., Yang Y., Li Q., Fan X., Zhao D., Liu Q., Zhang C. (2022). The Underlying Starch Structures of Rice Grains with Different Digestibilities but Similarly High Amylose Contents. Food Chem..

[7] Zhao Q., Selomulya C., Xiong H., Chen X.D., Ruan X., Wang S., Xie J., Peng H., Sun W., Zhou Q. (2012). Comparison of Functional and Structural Properties of Native and Industrial Process-Modified Proteins from Long-Grain Indica Rice. J. Cereal Sci..

[8] Wang Y., Xin Q., Miao Y., Zeng X., Li H., Shan K., Nian Y., Zhao D., Wu J., Li C. (2022). Interplay between Transglutaminase Treatment and Changes in Digestibility of Dietary Proteins. Food Chem..

[9] Wang H., Zhang Y., Yuan Z., Zou X., Ji Y., Hou J., Zhang J., Lu F., Liu Y. (2022). Crosslinking Mechanism on a Novel Bacillus Cereus Transglutaminase-Mediated Conjugation of Food Proteins. Foods.

[10] Shahid A., Haq M.A., Hussain N., Nawaz H., Xu J. (2025). Impact of Deep Eutectic Solvent Deamidation on the Solubility and Conformation of Rice Dreg Protein Isolate. Cereal Chem..

[11] Schlangen M., Ribberink M.A., Taghian Dinani S., Sagis L.M.C., van der Goot A.J. (2023). Mechanical and Rheological Effects of Transglutaminase Treatment on Dense Plant Protein Blends. Food Hydrocoll..

[12] Babiker E.E. (2000). Effect of Transglutaminase Treatment on the Functional Properties of Native and Chymotrypsin-Digested Soy Protein. Food Chem..

[13] Kim Y., Kee J.I., Lee S., Yoo S.-H. (2014). Quality Improvement of Rice Noodle Restructured with Rice Protein Isolate and Transglutaminase. Food Chem..

[14] Wu X., Chen X., Cai X., Wang S. (2024). Lipid-Lowering Activity and Underlying Mechanism of Glycosylated Peptide–Calcium Chelate Prepared by Transglutaminase Pathway. Food Front..

[15] Yang R., Wang C., Wu Z., Yu B., Yu X., Li C., Cao Y., Yuan C., Zhang Z., Zhao H. (2025). Structural and Gelation Properties of Soy Protein Isolates-Sesbania Gum Gels: Effects of Ultrasonic Pretreatment and CaSO_4_ Concentration. Int. J. Biol. Macromol..

[16] Liu Y., Wen Z., Sun J., Lu Y., Roopesh M.S., Cui L., Pan D., Du L. (2025). Cold Argon Plasma-Modified Pea Protein Isolate: A Strategy to Enhance Ink Performance and Digestibility in 3D-Printed Plant-Based Meat. Int. J. Biol. Macromol..

[17] Wang R.-X., Li Y.-Q., Sun G.-J., Wang C.-Y., Liang Y., Hua D.-L., Chen L., Mo H.-Z. (2023). Effect of Transglutaminase on Structure and Gelation Properties of Mung Bean Protein Gel. Food Biophys..

[18] Moguiliansky S., Friedman N., Davidovich-Pinhas M. (2025). The Effect of Transglutaminase on the Structure and Texture of Plant-Protein Based Bigel. Food Hydrocoll..

[19] Zhang J., Li T., Chen Q., Liu H., Kaplan D.L., Wang Q. (2023). Application of Transglutaminase Modifications for Improving Protein Fibrous Structures from Different Sources by High-Moisture Extruding. Food Res. Int..

[20] Li T., Zhang J., Hu A., Guo F., Zhou H., Wang Q. (2024). Effect of Transglutaminase and Laccase on Pea Protein Gel Properties Compared to That of Soybean. Food Hydrocoll..

[21] Udomrati S., Pantoa T., Sorndech W., Ploypetchara T. (2024). Investigation of Transglutaminase Incubated Condition on Crosslink and Rheological Properties of Soy Protein Isolate, and Their Effects in Plant-Based Patty Application. J. Food Meas. Charact..

[22] Chen Y., Lan D., Wang W., Zhang W., Wang Y. (2023). Effect of Transglutaminase-Catalyzed Crosslinking Behavior on the Quality Characteristics of Plant-Based Burger Patties: A Comparative Study with Methylcellulose. Food Chem..

[23] Zhang L.-D., Li L., Zhang Q., Wang Y.-Q., Liu Y., Yan J.-N., Lai B., Wang C., Wu H.-T. (2024). Impact of Plant and Animal Proteins with Transglutaminase on the Gelation Properties of Clam *Meretrix Meretrix* Surimi. Food Biosci..

[24] Song F., Gu Q., Liu L., Xu T., Li L., Yang Q., Lv L. (2025). Study on the Allergenicity and Gel Properties of Transglutaminase Cross-Linked Shrimp Myofibrillar Protein. Food Chem..

[25] Jiang S.-J., Zhao X.-H. (2010). Transglutaminase-Induced Cross-Linking and Glucosamine Conjugation in Soybean Protein Isolates and Its Impacts on Some Functional Properties of the Products. Eur. Food Res. Technol..

[26] Chen X., Zhao H., Wang H., Xu P., Chen M., Xu Z., Wen L., Cui B., Yu B., Zhao H. (2022). Preparation of High-Solubility Rice Protein Using an Ultrasound-Assisted Glycation Reaction. Food Res. Int..

[27] Hu A., Li L. (2022). Effects of Ultrasound Pretreatment on Functional Property, Antioxidant Activity, and Digestibility of Soy Protein Isolate Nanofibrils. Ultrason. Sonochem..

[28] Li T., Zhou J., Wu Q., Zhang X., Chen Z., Wang L. (2023). Modifying Functional Properties of Food Amyloid-Based Nanostructures from Rice Glutelin. Food Chem..

[29] Ince C., Condict L., Ashton J., Stockmann R., Kasapis S. (2024). Molecular Characterisation of Interactions between *β*-Lactoglobulin and Hexanal—An off Flavour Compound. Food Hydrocoll..

[30] Song C.-L., Zhao X.-H. (2014). Structure and Property Modification of an Oligochitosan-Glycosylated and Crosslinked Soybean Protein Generated by Microbial Transglutaminase. Food Chem..

[31] Zhao Q., Xiong H., Selomulya C., Chen X.D., Huang S., Ruan X., Zhou Q., Sun W. (2013). Effects of Spray Drying and Freeze Drying on the Properties of Protein Isolate from Rice Dreg Protein. Food Bioprocess Technol..

[32] Rodriguez-Espinosa M.E., Guevara-Oquendo V.H., He J., Zhang W., Yu P. (2025). Research Updates and Progress on Nutritional Significance of the Amides I and II, Alpha-Helix and Beta-Sheet Ratios, Microbial Protein Synthesis, and Steam Pressure Toasting Condition with Globar and Synchrotron Molecular Microspectroscopic Techniques with Chemometrics. Crit. Rev. Food Sci. Nutr..

[33] Korićanac M., Mijalković J., Petrović P., Pavlović N., Knežević-Jugović Z. (2025). Exploring Green Proteins from Pumpkin Leaf Biomass: Assessing Their Potential as a Novel Alternative Protein Source and Functional Alterations via pH-Shift Treatment. Bioresour. Bioprocess..

[34] Hu X., Zhao M., Sun W., Zhao G., Ren J. (2011). Effects of Microfluidization Treatment and Transglutaminase Cross-Linking on Physicochemical, Functional, and Conformational Properties of Peanut Protein Isolate. J. Agric. Food Chem..

[35] Lian Z., Yang S., Cheng L., Liao P., Dai S., Tong X., Tian T., Wang H., Jiang L. (2023). Emulsifying Properties and Oil–Water Interface Properties of Succinylated Soy Protein Isolate: Affected by Conformational Flexibility of the Interfacial Protein. Food Hydrocoll..

[36] Cao M., Liao L., Zhang X., Chen X., Peng S., Zou L., Liang R., Liu W. (2023). Electric Field-Driven Fabrication of Anisotropic Hydrogels from Plant Proteins: Microstructure, Gel Performance and Formation Mechanism. Food Hydrocoll..

[37] Alabi O.O., Annor G.A., Amonsou E.O. (2023). Effect of Cold Plasma-Activated Water on the Physicochemical and Functional Properties of Bambara Groundnut Globulin. Food Struct..

[38] Gorinstein S., Goshev I., Moncheva S., Zemser M., Weisz M., Caspi A., Libman I., Lerner H.T., Trakhtenberg S., Martín-Belloso O. (2000). Intrinsic Tryptophan Fluorescence of Human Serum Proteins and Related Conformational Changes. J. Protein Chem..

[39] Jiang S.-J., Zhao X.-H. (2011). Transglutaminase-Induced Cross-Linking and Glucosamine Conjugation of Casein and Some Functional Properties of the Modified Product. Int. Dairy J..

[40] Kang Z.-L., Yao P.-L., Xie J.-J., Li Y.-P., Ma H.-J. (2024). Effects of Low-Frequency Magnetic Field on Solubility, Structural and Functional Properties of Soy 11S Globulin. J. Sci. Food Agric..

[41] Tang C.-H., Sun X., Yin S.-W., Ma C.-Y. (2008). Transglutaminase-Induced Cross-Linking of Vicilin-Rich Kidney Protein Isolate: Influence on the Functional Properties and in Vitro Digestibility. Food Res. Int..

[42] Vanqa N., Mshayisa V.V., Basitere M. (2022). Proximate, Physicochemical, Techno-Functional and Antioxidant Properties of Three Edible Insect (*Gonimbrasia belina*, *Hermetia illucens* and *Macrotermes subhylanus*) Flours. Foods.

[43] Kutzli I., Weiss J., Gibis M. (2021). Glycation of Plant Proteins via Maillard Reaction: Reaction Chemistry, Technofunctional Properties, and Potential Food Application. Foods.

[44] Abdelraheem A.N., Hashim A.S., Ahmed M.E. (2010). Changes in Functional Properties by Transglutaminase Cross Linking as a Function of pH of Legumes Protein Isolate. Innov. Rom. Food Biotechnol..

[45] Yao X.-N., Dong R.-L., Li Y.-C., Lv A.-J., Zeng L.-T., Li X.-Q., Lin Z., Qi J., Zhang C.-H., Xiong G.-Y. (2024). pH-Shifting Treatment Improved the Emulsifying Ability of Gelatin under Low-Energy Emulsification. Int. J. Biol. Macromol..

[46] Dong C., Zhao J., Wang L., Jiang J. (2023). Modified Pea Protein Coupled with Transglutaminase Reduces Phosphate Usage in Low Salt Myofibrillar Gel. Food Hydrocoll..

[47] Sun Z., Zhang W., Zhang F., Chiu I., Li D., Wu X., Yang T., Gao Y., Zheng H. (2025). Physicochemical Modulation of Soy-Hemp-Wheat Protein Meat Analogues Prepared by High-Moisture Extrusion Using Transglutaminase. Food Res. Int..

[48] Tang C., Yang X.-Q., Chen Z., Wu H., Peng Z.-Y. (2005). Physicochemical and Structural Characteristics of Sodium Caseinate Biopolymers Induced by Microbial Transglutaminase. J. Food Biochem..

[49] Hiller B., Lorenzen P.C. (2009). Functional Properties of Milk Proteins as Affected by Enzymatic Oligomerisation. Food Res. Int..

[50] Velazquez-Dominguez A., Hennetier M., Abdallah M., Hiolle M., Violleau F., Delaplace G., Peres De Sa Peixoto P. (2023). Influence of Enzymatic Cross-Linking on the Apparent Viscosity and Molecular Characteristics of Casein Micelles at Neutral and Acidic pH. Food Hydrocoll..

[51] Dash D.R., Singh S.K., Singha P. (2024). Viscoelastic Behavior, Gelation Properties and Structural Characterization of Deccan Hemp Seed (*Hibiscus cannabinus*) Protein: Influence of Protein and Ionic Concentrations, pH, and Temperature. Int. J. Biol. Macromol..

[52] Spotti M.J., Martinez M.J., Pilosof A.M.R., Candioti M., Rubiolo A.C., Carrara C.R. (2014). Rheological Properties of Whey Protein and Dextran Conjugates at Different Reaction Times. Food Hydrocoll..

[53] Liang G., Chen W., Wang Z., Chu X., Zeng M., He Z., Goff H.D., Chen J. (2025). Hydrogel Structure of Soy Protein and Gelatin Dual Network Based on TGase Cross-Linking. Food Hydrocoll..

[54] Cain R.L., Webb I.K. (2025). Comparison of Partially Denatured Cytochrome c Structural Ensembles in Solution and Gas Phases Using Cross-Linking Mass Spectrometry. J. Am. Soc. Mass Spectrom..

[55] Kamiloglu S., Tomas M., Ozkan G., Ozdal T., Capanoglu E. (2024). In Vitro Digestibility of Plant Proteins: Strategies for Improvement and Health Implications. Curr. Opin. Food Sci..

[56] Tang C.-H., Li L., Yang X.-Q. (2006). Influence of Transglutaminase-Induced Cross-Linking on in Vitro Digestibility of Soy Protein Isolate. J. Food Biochem..

